# Dedication Oration, Greene Vardiman Black Memorial*Delivered August 7th in Chicago and reprinted from *Oral Health* for September, 1918.

**Published:** 1918-10

**Authors:** A. W. Thornton


### DEDICATION ORATION, GREENE VARDIMAN BLACK MEMORIAL*

BY A. W. THORNTON, D.D.S.

It would be the veriest hypocracy for any man to pretend that he did not look upon such a privilege as is mine today, as a very great honor, and it would be an insult to your intelligence to expect you to believe that such an occasion is one of the ordinary events of any man's life.

I appreciate the honor and feel the responsibility more deeply, perhaps, than any of you can imagine.

Any ordinary man might, perhaps, have some knowledge of the application of colors to a canvas, might possess some ability in freehand drawing, might understand the underlying principles of perspective, but such knowledge or such ability would not justify a person in attempting to paint a landscape or a portrait to hang in an art gallery with the world's masterpieces.

And so, it appears to me, the occasion that brings us together at this moment, demands the effort of a "workman that needeth not to be ashamed" to worthily deal with the subject which has been assigned me. Let me say to you in absolute frankness and in perfect honesty, that I feel totally incapable of measuring up to what you have a right to expect. But when your president asked me to undertake this duty, there was but one thing to do—accept.

And, if I fail adequately to express the love which we all felt for the man to whose life and labors this memorial is dedicated, let your own full hearts at this moment, be the measure of that appreciation, which my poor words must fall short of expressing.

---

*Delivered August 7th in Chicago and reprinted from *Oral Health* for September, 1918.

As I tried during the past weeks, to think of the exercises in which we were to take part today, there came to my mind again and again, the words of King David, spoken three thousand years ago of Abner, "A prince and a great man is fallen." The term "a great man," is frequently heard, and yet, to what a comparatively few men, can the words be fitly applied.

In law, Moses stands pre-eminent, for the simple reason that the moral law, the Ten Commandments, stands today as it stood three thousand five hundred years ago, the very foundation principle of national as well as of individual greatness.

In sociology, the world has seen but one perfect example—The Man of Nazareth. In a few sentences, He laid on bed rock, the basic principles of ethical relationships between man and man, and between nation and nation, and the violation of these principles by a powerful European nation, is the cause of the terrific struggle now convulsing the world. Let me quote just a sentence or two, embodying some of His foundation principles:

"Whatsoever ye would that men should do to you, do ye even so unto them likewise." "I came not to be ministered unto, but to minister." "Let him that would come after me, deny himself." "He that loseth his life shall find it, shall keep it unto life eternal."

Read today the biography of the world's great men and you will find a striking similarity in life and thought and purpose in all of them. What man or woman can possibly attain to greatness and persistently violate any one of the Ten Commandments? Would you call any man great who would attempt to deal with his fellowman as he would not wish a fellowman to deal with him? Could a man by any stretch of imagination, be called great, who was always receiving the ministrations of others in selfish enjoyment? What man today has the greatest chance of being classed with the world's great men? Is it not the man who has learned most perfectly, the lesson of self-denial?

In the world's great cataclysm at the present time, who are the men whose names and whose memories are being enshrined in the hearts of all right thinking persons? Are they not the men who are losing their lives in order that national and individual lives may find a chance to express themselves along God-given lines? And was it not along the lines that I have just indicated that the life of the man whose memory we have met to honor, found expression?

Think of the work accomplished in a single life-time. It was fundamental in character, it was stupendous in volume. When his own operations, as well as the operations of other men failed, he sought the reason of the failure and the means by which failure might be avoided, and he gave to the profession, scientific methods of operation which shall bless humanity while time lasts.

There is a question that must be ringing in the mind of every man here present. It is this: Why is it that men such as Dr. Black are able to accomplish so much, while most of us do so little? Is the difference between such men and the rest of us simply one of mental endowment? Are these men mental giants and the rest of us mental pigmies? No. I think not. What then is the reason? Is this the explanation? Only a few men in each generation are capable of sustained effort and this, perhaps, is the secret of all true greatness. Longfellow has expressed that thought in these words:

"We have not wings, we can not soar,
But we have feet to scale and climb
By slow degrees, by more and more
The cloudy summits of our time."

"The mighty pyramids of stone
That wedge-like cleave the desert airs,
When nearer seen and better known,
Are but gigantic flights of stairs."

And is this not the secret of the unparalleled success of him whose memory and whose life-work we delight to honor? Do you know that it takes fifteen pages of ordinary magazine size, in very small type, simply to give the titles of Dr. Black's contributions to the profession, of which he was the most distinguished member? The desire to attain to his own full stature must have taken possession of him even in early life.

Some years ago, I attended with Dr. Black, and a number of other men from Chicago, the fiftieth anniversary of the St. Louis Dental Association. At that meeting, Dr. Black showed some lantern slides of sections of teeth that he had made years before, by hand with a pen and India ink, before such slides were made by photography, and they were as accurate and almost as delicate as those made by the scientific apparatus now available.

But the books which he wrote, the contributions which he made to so wide a field in science, the teaching he did in a great university, these are not the things that call out today, this voluntary expression of gratitude and esteem. We admire the ability which enabled him to overcome, where smaller men must have met with failure. We acknowledge the debt we owe, because we are able to accomplish things which would have been altogether impossible had he not blazed the pathway to success.

We feel keenly our own lack of attainment when we think of him "in labors more abundant," but, we loved him—not for what he did, but for what he was.

Many of the men taking part in this exercise today, have a mental picture of him as they knew him best. Some will think of him as they met him at professional gatherings and the outstretched hand, the genial smile, the kindly word, seem to forge again the link that was broken, and they wonder if it can be indeed true that the "silken cord has been loosed." Others, especially the members of faculties from other schools, will have a vision of him in his own school, as the hearty, unassuming welcome was extended. Others, perhaps, by far the largest number, will think of him as he walked through the infirmary, dropping a hand on a shoulder here, offering a word of advice there, giving a little help with an operation some other place, and as he did so, many a difficult operation became easier, many a problem was more readily solved, many a downhearted student felt that a veritable benediction had come "by the laying on of hands."

But why, may I ask, are we today, dedicating and unveiling a memorial to Dr. Black? Why in all ages and in every country, have monuments been raised to great and good men?

"Can storied urn or animated bust,
Back to its mansion call the fleeting breath?
Can honor's voice provoke the silent dust?
Or flattery soothe the dull, cold ear of death?"

We all know that this can mean nothing to him, who so short a time ago joined the great company of those whom "we have loved long since and lost awhile." Why then do we do these things?

Two reasons, perhaps, actuate us: The first, to show to the world and to his immediate dear ones, that we loved the man, and appreciate the life-work fraught with such momentous importance to all future generations; and in the second place, to inspire others to lead lives devoted, as his was, to the amelioration of suffering and to the extension of scientific education and individual culture, the only foundation upon which it is possible to rear the superstructure of national and individual greatness, happiness and prosperity.

More than three thousand years ago, that greatest of all questions was asked: "If a man die, shall he live again?" It has never been answered as we would answer or prove a proposition in Euclid so that we might write after the answer, "Which was required to be demonstrated."

But strange indeed, would be our conception of creation or evolution, whichever you wish, if having ears, there were no songs of birds or laughter of children, no strains of sweet music nor articulate sounds of loving voices. It would be strange, would it not, if having eyes to see, there were no rosy morns or glowing sunsets, no green valleys nor snow-capped mountains, no mountain torrent flashing its myriad of crystals in the sun or placid lake reflecting back the softened rays of a harvest moon, "no sky and flowers and trees."

So, I believe that in some way, the greatest yearning of the human soul, its capability for love and service and companionship will be satisfied "when the golden bowl is broken, when the pitcher is broken at the fountain, when the wheel is broken at the cistern and the spirit returns unto God who gave it."

To have known a man like Dr. Black, to have enjoyed his friendship, to have felt the warmth of his social nature, to have feasted mentally so often and so bounteously on the satisfying mental pabulum of his production, is to intensify and make more real the belief that "when the earthly house of this tabernacle is dissolved, we have a building of God, an house not made with hands, eternal in the Heavens."

In the Genesis account of creation, it is said that God created man in His own image and likeness. It is in the lives of such men as Greene Vardiman Black, that the eternal, the infinite, the loving nature of God is most clearly discernable. In the early ages, the passing of such men was spoken of in some such words as these: "Having served his day and generation, he has fallen on sleep."

Could words be truer of any man than of Dr. Black? Having served his day and generation, full of years and of honor, loved most by those who knew him best. "He has fallen on sleep." May "a double portion of his spirit" fill all our hearts.

Perhaps Dr. Black's philosophy of life, life here, life hereafter, might be summed up in the following words:

"For me to have made one Soul,
The better for my birth:
To have added but one flower
To the garden of the earth.

"To have struck one blow for truth,
In the daily fight with lies:
To have done one deed of right
In the face of calumnies.

"To have sown in the souls of men
One thought that will not die,
To have been a link in the chain of life,
'Twill be Immortality."

